# Applications and properties of computationally designed *de novo* proteins

**DOI:** 10.1063/4.0000965

**Published:** 2025-10-27

**Authors:** Martin Pacesa

**Affiliations:** EPFL, Lausanne, Vaud, Switzerland

## Abstract

Computational protein design is emerging as a powerful technique for creating novel protein tools with applications in structural biology, diagnostics, and therapeutics. Recent advances, particularly deep learning methods such as AlphaFold2, have significantly accelerated our ability to design stable, arbitrary protein folds with high experimental success rates. In this talk, we will explore the biophysical properties and potential applications of these *de novo* designed proteins, focusing specifically on addressing challenges associated with designing functional proteins that mediate precise biological interactions. To overcome these challenges, we developed BindCraft, an open-source, automated pipeline for *de novo* protein binder design. BindCraft achieves high experimental success rates (10-100%) and generates binders with nanomolar affinity without requiring extensive screening or experimental optimization—even for targets without previously characterized binding sites. We showcase this by designing binders against diverse targets, including cell-surface receptors, common allergens, and complex multi-domain nucleases such as CRISPR-Cas9. Additionally, we demonstrate practical applications by significantly reducing allergen binding in patient-derived samples and redirecting AAV capsids for targeted gene delivery. This work highlights how computationally designed proteins can serve as versatile tools in structural biology, opening new opportunities for investigating biological mechanisms and facilitating advanced structural studies.

